# Large Right Atrial Myxoma Presenting As Bilateral Pulmonary Embolism

**DOI:** 10.7759/cureus.15889

**Published:** 2021-06-24

**Authors:** Aref Obagi, Dhaval Desai, Usman Mazahir, David Johnson, Lance Berger

**Affiliations:** 1 Cardiology, Jersey Shore University Medical Center, Neptune, USA; 2 Cardiovascular Disease, Jersey Shore University Medical Center, Neptune, USA; 3 Internal Medicine, Trinitas Regional Medical Center, Elizabeth, USA; 4 Pulmonary and Critical Care Medicine, Jersey Shore University Medical Center, Neptune, USA; 5 Cardiac Surgery, Jersey Shore University Medical Center, Neptune, USA

**Keywords:** benign cardiac tumor, cardiac tumor in adults, right atrial myxoma, acute pulmonary embolism, mass resection

## Abstract

Myxoma is a rare benign tumor of the heart. Cardiac myxomas are the most common primary cardiac tumor in adults, commonly found within the left atrium. It can occur at any age and is more common in females than males. This case report aims to identify the clinical symptoms of cardiac myxoma, which can be life-threatening if neglected. Here, we present the case of a 30-year-old female with past smoking history. For the past three to four weeks before this hospitalization, her symptoms worsened including shortness of breath with exertion, dry cough, and pleuritic chest pain. Outpatient treatment with antibiotics and nebulizers did not relieve her symptoms. She went to the emergency room and underwent computed tomography of the chest with contrast showing bilateral lower lobe pulmonary emboli and a large mass in the right atrium. Intravenous unfractionated heparin was initiated. A transthoracic echocardiogram confirmed a 3.76 cm × 4.95 cm mass in the right atrium. The patient underwent surgical resection of the right atrial mass the following day and was discharged four days later in a stable condition. Pathology of the mass confirmed atrial myxoma.

## Introduction

Primary cardiac tumors are extremely rare, with an incidence of less than 0.1% found in 12,000 autopsies [1]. Primary cardiac tumors are less common than secondary (metastatic) tumors, with incidence for primary tumors of 0.056% versus 1.23% for secondary tumors found in 12,485 autopsies [2]. Cardiac myxomas are more common in the left atrium as opposed to the right atrium [3]. The mean age of patients diagnosed with cardiac myxoma is 50 years, occurring more commonly in females [4].

## Case presentation

A 30-year-old woman with a past medical history of tobacco use presented to our facility with worsening shortness of breath on exertion, dry cough, and pleuritic pain. Outpatient treatment with antibiotics and nebulizers did not relieve her symptoms. She then presented to the emergency room (ER). In the ER, she was in no apparent distress; her blood pressure was 122/75 mmHg, heart rate 90 beats per minute, respiratory rate 18 breaths per minute, temperature 98.2°F, and pulse oximetry was 95% on room air. Heart and lung examinations were normal.

**Figure 1:**
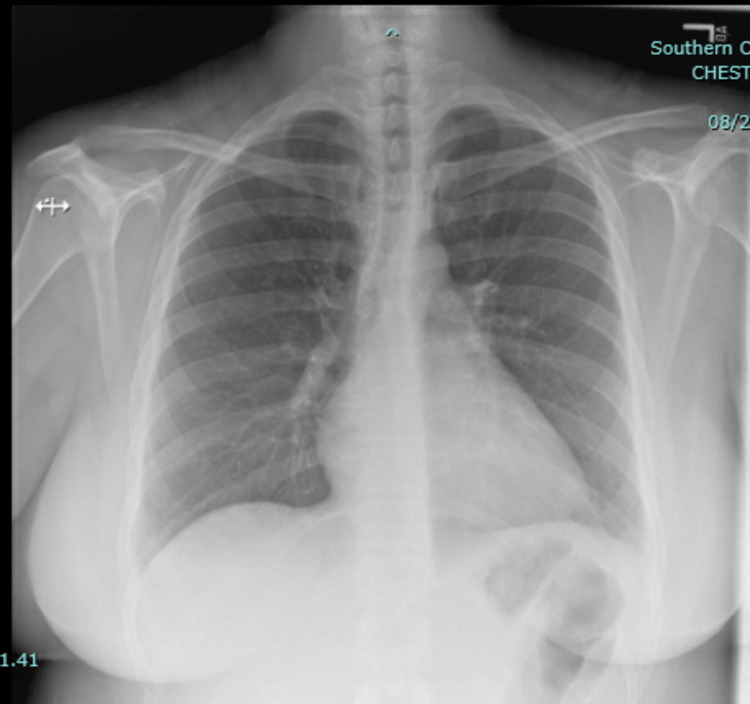
Chest X-ray showing normal lungs, with no consolidation or pleural effusion and normal cardiac silhouette.

Laboratory tests revealed white blood cell count of 15.6 × 10^3^/uL (reference range: 4.5-11.0 × 10^3^/uL), hemoglobin 12.1 g/dL (reference range: 13.2-17.5 g/dL), platelet count 410 × 10^3^/uL (reference range: 140-450 × 10^3^/uL), blood urea nitrogen 19 mg/dL (reference range: 5-25 mg/dL), creatinine 0.95 mg/dL (reference range: 0.61-1.24 mg/dL), and troponin 0.01 ng/mL (reference range: <0.04 ng/mL). Chest X-ray showed no evidence of pneumonia or pleural effusion (Figure 1).

**Figure 2:**
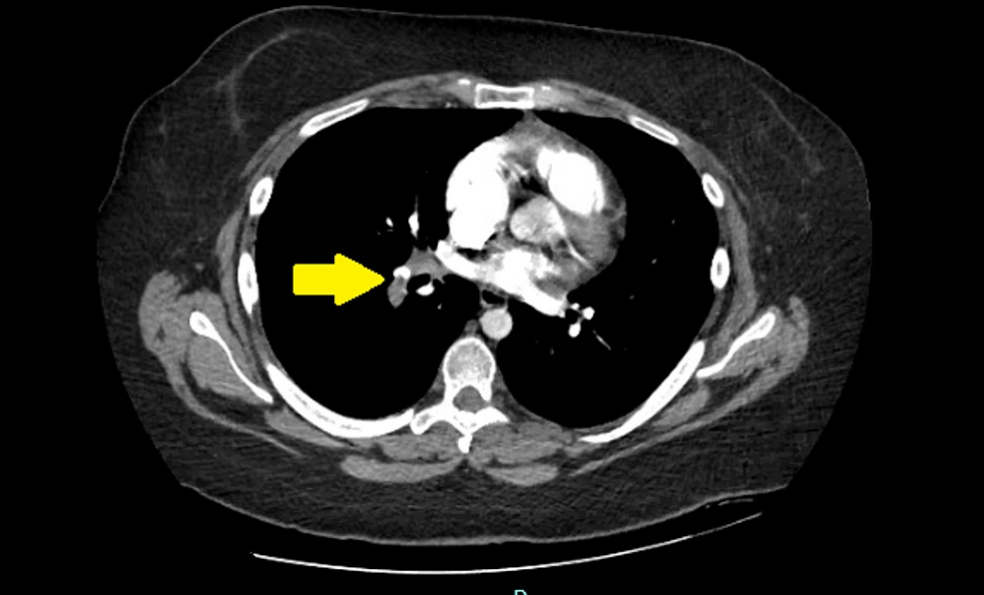
CT of the chest with contrast showing a small right lower lobe pulmonary embolism (yellow arrow). CT: computed tomography

**Figure 3:**
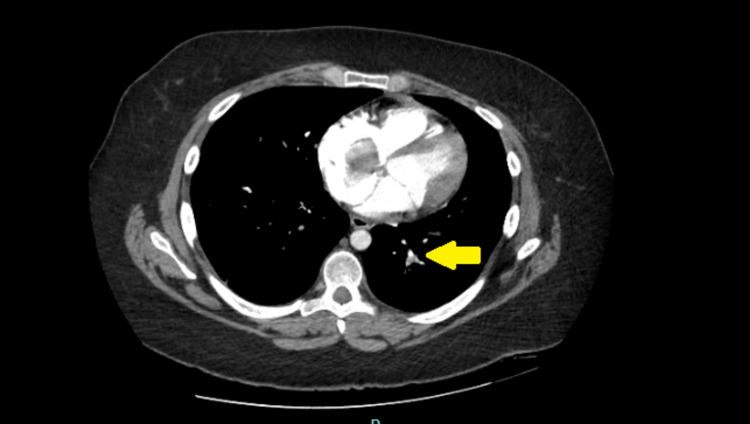
CT of the chest with contrast showing a small left lower lobe pulmonary embolism (yellow arrow). CT: computed tomography

**Figure 4:**
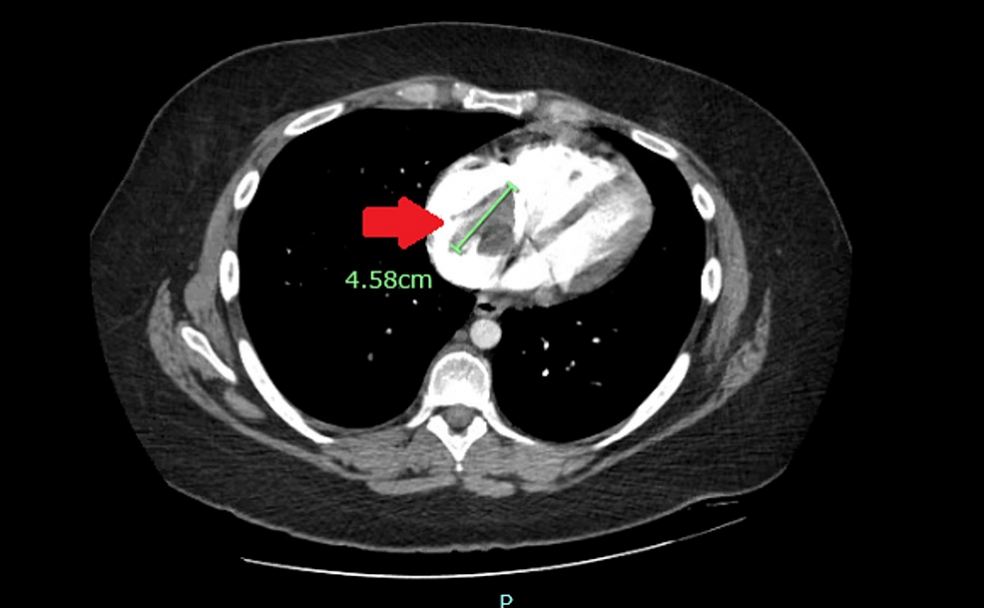
CT of the chest with contrast showing a 4.5 cm mass in the right atrium (red arrow). CT: computed tomography

Given the persisting symptoms despite outpatient medical management and clear chest X-ray, she underwent computed tomography (CT) of the chest with contrast showing bilateral lower lobe pulmonary emboli (Figures 2, 3) and a 4.5 cm mass in the right atrium (Figure 4).

**Figure 5:**
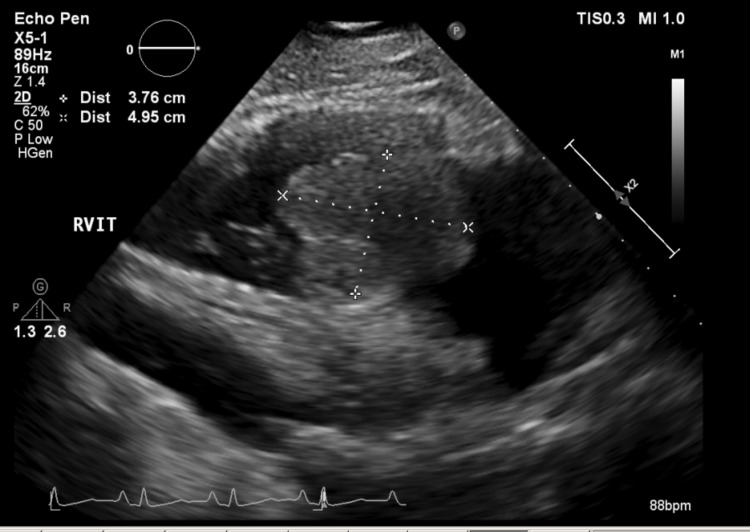
Transthoracic echocardiogram with RV inflow window showing a large mass measuring 3.7 × 4.9 cm. RV: right ventricular

**Figure 6:**
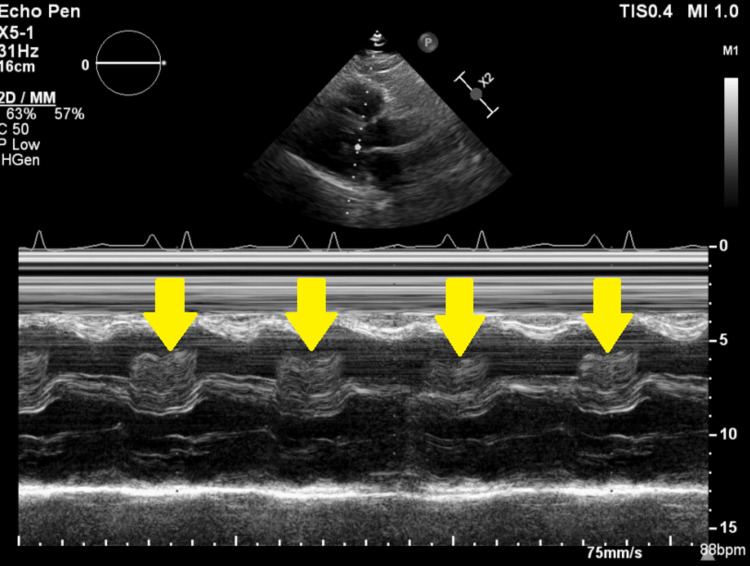
M-mode recording demonstrating the mass prolapsing into the RV during diastole “tumor plop” (yellow arrow). RA: right atrium

Intravenous unfractionated heparin was started. A subsequent transthoracic echocardiogram confirmed a 3.76 cm × 4.95 cm mass in the right atrium (Video 1, Figures 5, 6).

**Video 1:** Transthoracic echocardiogram with RV inflow window showing the large and highly mobile mass in the RA protruding through the tricuspid valve and obstructing the RV inflow. RA: right atrium; RV: right ventricular

**Figure 7:**
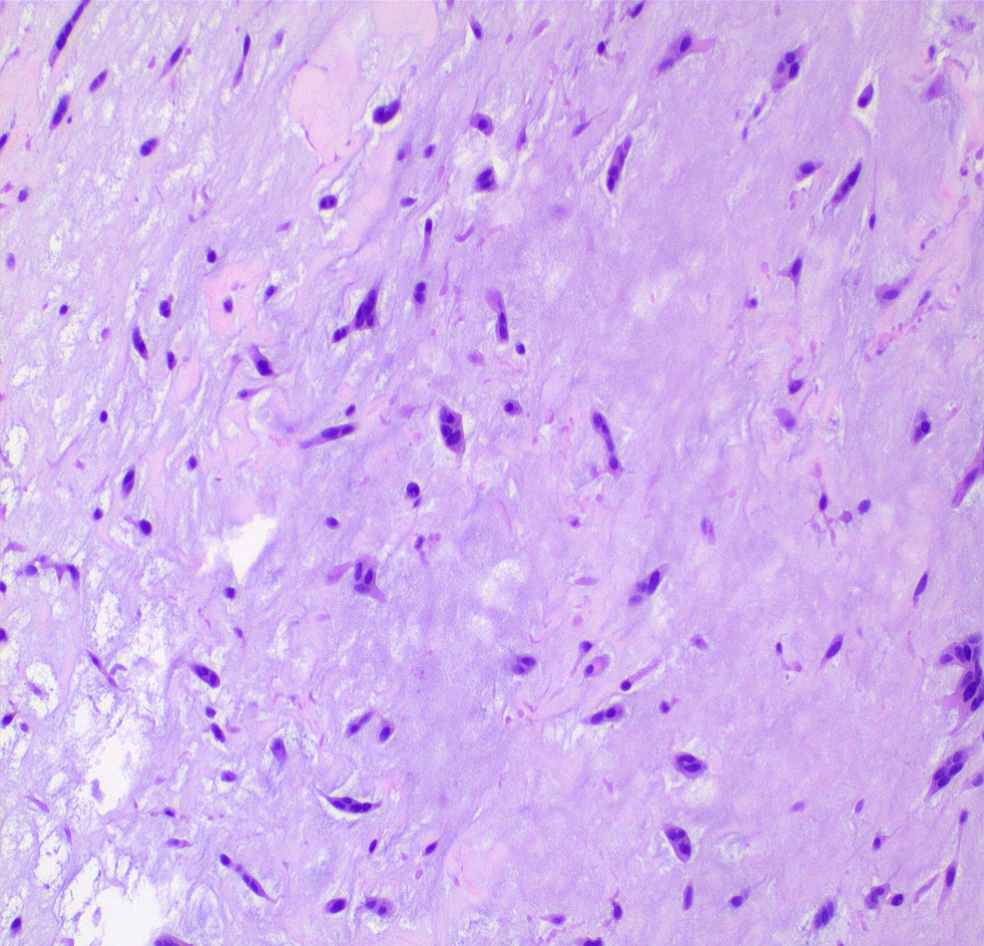
Stellate-shaped myxoma cells. H&E stain (original magnification ×400). H&E: hematoxylin and eosin

A discussion was held with the cardiothoracic surgery team. The patient underwent surgical excision of the right atrial mass the following day. An intraoperative transesophageal echocardiogram revealed the massive right atrial mass with a stalk attached to the interatrial septum (Video 2). Pathology of the mass confirmed right atrial myxoma (Figure 7). The patient was discharged four days later to a rehab facility in a stable condition.

**Video 2:** Transesophageal echocardiogram four-chamber view showing the large right atrial mass attached to the interatrial septum and prolapsing through the tricuspid valve into the RV and compressing the interventricular septum into the LV during diastole. RA: right atrium; LA: left atrium; RV: right ventricle; LV: left ventricle

## Discussion

Atrial myxomas are the most common benign primary cardiac tumors with a prevalence of around 0.01% [1]. Most cases occur between the third and sixth decade of life, with a high prevalence among females. Although most myxomas arise from the border of the fossa ovalis in the left atrium, rarely they can emerge from the right atrium, as in our case [5]. Morphologically, they are pedunculated smooth polypoid round or oval structures with their mobility dependent on their attachment site and stalk length. Clinical presentation can vary from asymptomatic to systemic symptoms to sudden cardiac death [6]. Due to the nonspecific presentation, diagnosis can be challenging. Left-sided myxomas with racemose structure and size over 5 cm in diameter are more likely to produce symptoms [7]. Myxoma-related symptoms are usually produced by mechanical interference with valvular function, leading to stenotic or regurgitant lesion or embolization due to their highly vascular and friable nature.

For diagnostic evaluation, a transthoracic echocardiogram is widely available and provides essential information such as tumor location, size, mobility, and attachment. Color Doppler can provide further details regarding hemodynamic consequences. Transesophageal echocardiogram has 100% sensitivity and is more specific compared to transthoracic echocardiogram. Cardiac magnetic resonance imaging delineates the point of attachment most precisely with the postsurgical correlation of 83% [8].

Surgery is the treatment of choice, with perioperative mortality around 2.2%. Postoperative atrial arrhythmia has been reported in 26% of cases [9]. In sporadic cases, 2-5% of patients can have recurrent myxoma, whereas 12% in familial cases and 22% in complex atrial myxomas. Currently, there is no consensus on the adequate follow-up interval for recurrence or for screening family members.

## Conclusions

This case emphasizes the varying presentation of atrial myxoma and the diagnostic challenge it may present. Echocardiography is a widely available noninvasive tool that can identify the tumor and should be performed early in patients with suspicion. Surgical resection is usually curative with an excellent success rate but is associated with a risk of developing atrial arrhythmias. Currently, there is a lack of guidelines on determining the optimal time for surgical intervention and decision to initiate anticoagulation therapy as there is an increased risk of thrombotic complication in addition to the embolization of the tumor mass. Further research is needed for determining the optimal follow-up duration and frequency for tumor recurrence, primarily when it occurs in younger patient populations and at atypical locations.
